# Oscillation Suppression in the Sense Mode of a High-Q MEMS Gyroscope Using a Simplified Closed-Loop Control Method

**DOI:** 10.3390/s18082443

**Published:** 2018-07-27

**Authors:** Qiang Shen, Xinpeng Wang, Yixuan Wu, Jianbing Xie

**Affiliations:** 1 Research & Development Institute, Northwestern Polytechnical University, Shenzhen 518057, China; xpwang@mail.nwpu.edu.cn (X.W.); wuyixuan@mail.nwpu.edu.cn (Y.W.); xiejb@nwpu.edu.cn (J.X.); 2 MOE Key Laboratory of Micro and Nano Systems for Aerospace, Northwestern Polytechnical University, Xi’an 710072, China

**Keywords:** MEMS gyroscope, sense-mode oscillation suppression, control circuit

## Abstract

The oscillation of the sense mode of the micro-machined Coriolis vibratory gyroscope (MCVG) with high quality factor (*Q*) is analyzed in this study and the corresponding force feedback control scheme is presented to suppress this oscillation. The controller consists of integrator and some filters, instead of the common but complicated demodulation and remodulation modules. Compared with using no oscillation suppression scheme, the proposed simplified oscillation suppression control scheme can achieve an improvement of the sense mode of the MCVG. The inband spectrum ripple of the angular rate output are improved from 51.4 dB to 4.23 × 10^−4^ dB. Correspondingly, these two performance parameters are improved by 370.4 and 186.2 times, which are higher than two orders of magnitude, respectively. Bias stability is improved from 9.72 deg/h to 2.5 deg/h. Test results prove that the proposed control scheme is effective in suppressing the oscillation.

## 1. Introduction

The micro-machined Coriolis vibratory gyroscope (MCVG) has been widely used in many fields, including for motor vehicles, robotics, and smartphones, because of its low cost and power, and high yield rate. However, its low performance limits further high-end applications of the MCVG. One important factor limiting its performance is the inevitable zero-rate output drift of the gyroscope, also known as the bias drift [1].

In order to reduce the bias and suppress bias drift, the bias sources resulting from various aspects have been analyzed, and corresponding control schemes have also been discussed in the published literature. First, the drive voltage will be coupled to the sense mode through the parasitic capacitances between the wire bonding pads, wires, proof mass, substrate, etc. [2,3,4] Researchers have aimed to reduce this in either the microfabrication process [5] or circuit design based on the trans-impedance amplifiers [6]. Second, the phase error in the circuits will inevitably result in an incomplete demodulation, which is also an important bias source [7,8,9,10]. This error is suppressed by selecting the reference phase of the drive-mode control circuits. Third, the mechanical movement in the drive mode will be coupled to the sense mode because both modes cannot be ensured to be perfectly orthogonal by current fabrication technologies, which is also known as the quadrature error [2,11,12]. The quadrature error of the MCVG has usually been suppressed by adaptive control, sigma-delta demodulator scheme, or demodulation and modulation scheme [6,13,14,15,16,17,18,19,20].

Currently, the high-vacuum packaging technology is an efficient method to improve the performance of the MCVG. It can suppress some bias sources by increasing the quality factor (*Q*) of the MCVG [21,22,23,24]. Nevertheless, the MCVG, as a typical two-order mass-damping-stiffness system, almost works in a critical damping state with increasing *Q*. Especially, when the sense-mode resonant frequency further approaches the drive-mode frequency with the aim of achieving higher signal noise ratio (SNR), the harmful oscillation of the sense-mode of the MCVG will be inevitably induced by slight external disturbances or the drive-mode vibration in small bandwidth. This will lead to a drastic fluctuation of the bias and severe performance deterioration. In our experiments, for a gyroscope with a *Q* of 30,000 in the sense mode with small bandwidth, the oscillation can generate an alternating current (AC) signal of 230 millivolts, which will cause the bias to fluctuate over time, severely deteriorating the gyroscope performance. Some researchers have designed special mode-matched schemes to improve the bias stability and performance of the MCVG [15,20]. In these schemes, performance seriously depends on the phase accuracy of the modulation and demodulation modules of the sense mode. Currently, a part of these items has been confirmed to contribute to this phase, such as quadrature error, feedthrough coupling, and even thermal drift of the circuit components. Nevertheless, other potentially unknown contribution items have not been confirmed yet. Therefore, it is not easy to provide accurate phase confirmation to improve performance, which also drove us to design an alternative control scheme in the sense mode to suppress the oscillation. In this work, the oscillation of the sense mode is first analyzed in a typical symmetrical MCVG. Then, based on the proposed combination scheme of integrator and filter, a simplified closed-loop control method without demodulation or remodulation modules is presented to suppress the oscillation.

This paper is organized as follows. In Section 2, the oscillation of the sense mode is described and analyzed. In Section 3, a simulation of the oscillation suppression method is presented. In Section 4, the gyroscope with and without oscillation suppression is compared. Finally, conclusions are presented in Section 5.

## 2. Analysis of Oscillation of Sense Mode

**Figure 1:**
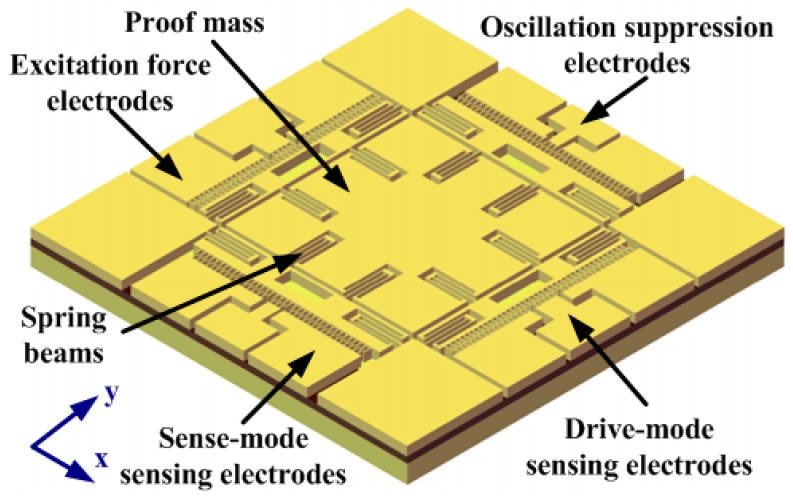
Schematic representation of the micro-machined Coriolis vibratory gyroscope (MCVG).

In this work, a completely symmetrical *Z*-axis gyroscope was selected as the controlling object, as shown in Figure 1. The detailed operation principles of the gyroscope are omitted here, as they were described in our previous work [24].

**Figure 2:**
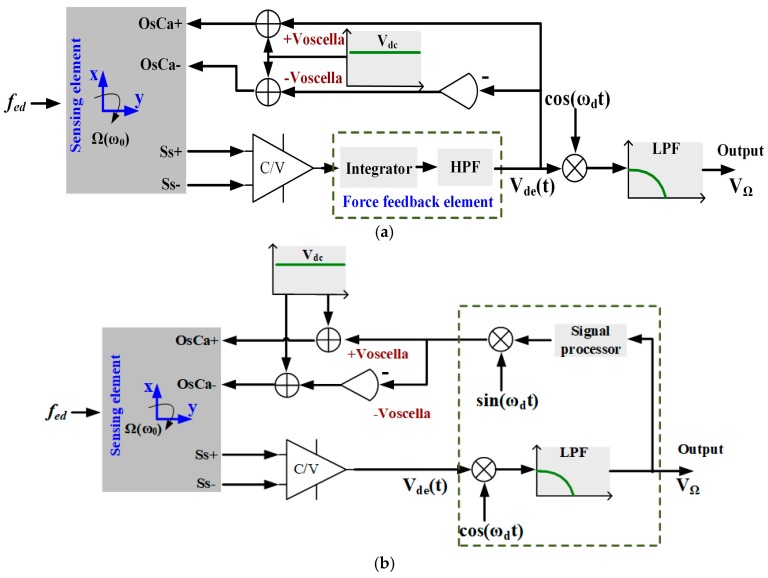
Schematic comparison of (**a**) the proposed force feedback control method and (**b**) the traditional method for the MCVG.

According to the schematic representation of the gyroscope, the angular rate detection electronics consists of the C/V sensing element of the gyroscope and the proposed force control element marked in the dotted box, as shown in Figure 2a. To be compared with the proposed scheme, the traditional method is also presented in a dotted box in Figure 2b [20]. Signals cos(*ω_d_t*) and sin(*ω_d_t*) belong to the demodulation and modulation modules, respectively.

In the angular rate detection electronics, when a random disturbance is applied to the gyroscope, the oscillation of the sense mode will occur easily at the resonance frequency *ω_s_* because of the high *Q* of the gyroscope, which is caused by the high-vacuum packaging. The random disturbance signal can be assumed as a cosine signal and its expression is written as

(1) $$e\left(t\right)=f_{ed}\cos\left(2\pi f_{s}t+\xi \right)\;$$

where *f_ed_* is the amplitude of the external disturbance, *ξ* is the phase offset, and *ω_s_* = 2π*f_s_* is the sense-mode resonance frequency. The transfer function from the external disturbance force to the oscillation displacement of the sense mode could be written as

(2) $$H_{gy}\left(s\right)=\frac{Y_{so}\left(s\right)}{f_{ed}\left(s\right)}=\frac{1}{m_{s}\left(s^{2}+\frac{\omega_{s}}{Q_{s}}s+\omega_{s}{}^{2}\right)}\;$$

where *s* = *jω_s_* and *m_s_* is the mass of the sense mode. Displacement of the oscillation can be written as

(3) $$y_{so}\left(t\right)=\frac{f_{ed}Q_{s}}{m_{s}\omega_{s}{}^{2}}\cos\left(\omega_{s}t+\xi +\frac{\pi }{2}\right)\;$$

From Equation (3), it can be seen that the amplitude of the oscillation is mainly dominated by the disturbance force *f_ed_* and the sense-mode quality factor *Q_s_*. In Figure 2, this displacement is directly picked from the Ss+ and Ss− ports of the sensing element. After the interface circuits, the oscillation voltage can be expressed as

(4) $$V_{de}\left(t\right)=\frac{f_{ed}Q_{s}}{m_{s}\omega_{s}{}^{2}}G_{y}^{V_{de}}\cos\left(\omega_{s}t+\frac{\pi }{2}+\xi +\eta \right)\;$$

where *η* and $G_{y}^{Vde}$ are very small phase shift and gain from the displacement *y_so_(t)* to the input *V_de_(t)* of the demodulator including the proposed integrator and filter, respectively. Subsequently, the signal is demodulated by the reference cosine signal cos(*ω_d_t*) and low-pass filtered to obtain the output voltage *V*_Ω_, which is written as

(5) $$V_{\Omega }\left(t\right)=\frac{f_{ed}Q_{s}G_{y}^{V_{de}}}{2m_{s}\omega_{s}{}^{2}}\cos\left(\left(\omega_{s}-\omega_{d}\right)t+\xi +\eta +\frac{\pi }{2}\right)\;$$

where the gyroscope output *V*_Ω_ generated by the oscillation is still a cosine signal with an oscillation frequency |*ω_s_* − *ω_d_*|.

**Figure 3:**
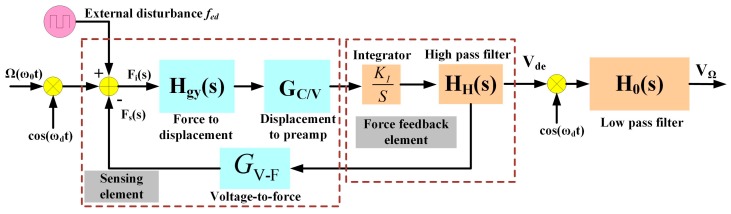
Transfer function of the sense-mode force feedback control system.

In the sense-mode force feedback loop, the output signal of the interface circuit module is fed into the controller consisting of the integrator and high-pass filter (HPF) located in the forward path. After the controller, the AC voltage *V_oscella_* is obtained and differentially applied on the oscillation suppression electrodes *OsCa+* and *OsCa−* in the sense mode with a fixed *V_dc_* bias voltage to generate the error suppression force. This force is in anti-phase with the external disturbance force *f_ed_*. Figure 3 shows the transfer function of the proposed sense-mode force feedback control system.

The open loop transfer function of the control system can be written as

(6) $$E\left(s\right)=\frac{F_{s}(s)}{F_{ed}(s)}=\frac{K_{I}}{s}H_{gy}\left(s\right)\cdot G_{C/V}\cdot H_{H}\left(s\right)\cdot G_{V\_F}\;$$

where *G_C/V_* is the gain of the displacement-to-voltage interface circuit module; *K_I_* is the gain of the proposed integrator; *E*(*s*) is dominated by the integrator and HPF characteristic, which are used to suppress the noise and keep the amplitude-frequency response constant within the frequency domain (*ω_d_* − *ω*_Ω_, *ω_d_* + *ω*_Ω_), where *ω_d_* is the resonance frequency of the drive mode of the gyroscope, and *ω*_Ω_ = 2π*f*_Ω_ is the working frequency of the input angular rate. *H_H_*(*s*) is the transfer function of the HPF and it is written as

(7) $$H_{H}\left(s\right)=\frac{s}{s+\omega_{c}}\;$$

where *ω_c_* = 2π*f_c_* is the cut-off frequency of the HPF (*ω_c_* is slightly smaller than *ω_d_* − *ω*_Ω_ to satisfy the dynamic response to the angular rate). The closed-loop transfer function of the control system at the resonance frequency *ω_d_* is written as

(8) $$Y\left(s\right)=\frac{H_{gy}\left(s\right)\cdot G_{C/V}K_{I}H_{H}\left(s\right)}{s\left(1+E\left(s\right)\right)}\;$$

In the closed-loop transfer function *Y*(*s*), the HPF and integrator are the strategic components that weaken the amplitude of the system at the frequency with *f_s_*. In addition, the inhibiting effect is illustrated through a simulation analysis in Section 3.

According to the Coriolis principle, frequency *ω*_Ω_ of the angular rate Ω is demodulated by frequency *ω_d_* of the drive-mode velocity $\dot{X}$, where *X* is the amplitude of the drive-mode displacement. When the angular rate Ω(*ω_0_t*) is input, *V_de_* can be written as

(9) $$V_{de}\left(j\omega_{d}\right)=m_{s}X\omega_{d}\left[\Omega \left(j(\omega_{0}+\omega_{d})\right)+\Omega \left(j(\omega_{0}-\omega_{d})\right)\right]\frac{H_{gy}\left(j\omega_{d}\right)\cdot G_{C/V}K_{I}H_{H}\left(j\omega_{d}\right)}{j\omega_{d}\left(1+E\left(j\omega_{d}\right)\right)}\;$$

After *V_de_*(*jω_d_*) is demodulated by *cos*(*ω_d_t*) and low-pass filtered by *H_0_*(*s*), of which cut-off frequency is slightly larger than *ω*_Ω_, the angular rate output signal *V*_Ω_ can be derived as

(10) $$V_{\Omega }\left(j\omega_{0}\right)=\frac{1}{2}m_{s}X\Omega \left(j\omega_{0}\right)\frac{H_{gy}G_{C/V}K_{I}H_{H}}{1+E}H_{0}\;$$

where *H_gy_*, *H_H_*, and *E* are gains of the sensing element, proposed filter, and *E*(*s*) at the resonance frequency *ω_d_*, respectively; *H*_0_ is the gain of *H*_0_(*s*) at the frequency *ω*_Ω_. Therefore, according to the size of *V*_Ω_, the angular rate input signal Ω(*ω_0_t*) could be calculated.

## 3. Simulation Analysis

To quantitatively illustrate the oscillation suppression of the sense mode of the high-*Q* MCVG, a SIMULINK model was constructed based on Figure 3 and the corresponding analysis. First, according to Equation (6), the simulation of the open-loop transfer function *E*(*s*) of the MCVG was performed. The corresponding time-domain and frequency-domain characteristics caused by the external vibration were analyzed. The simulation parameters are listed in Table 1. Subsequently, according to Equation (8), the simulation analysis of the closed-loop transfer function *Y*(*s*) was performed to verify the effectiveness of the proposed method. In order to compare them with the open-loop characteristics, the time-domain and frequency-domain characteristics were also analyzed. The corresponding closed-loop simulation parameters and comparison results are listed in Table 2 and Table 3, respectively.

**Figure 4:**
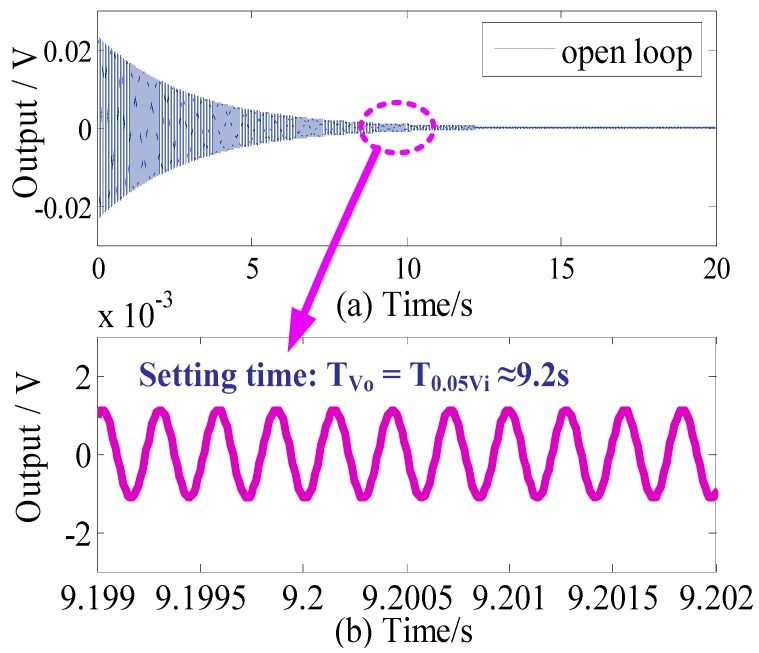
(**a**) Open-loop time-domain response of the sense mode and (**b**) enlarged view in 9.2 s.

**Figure 5:**
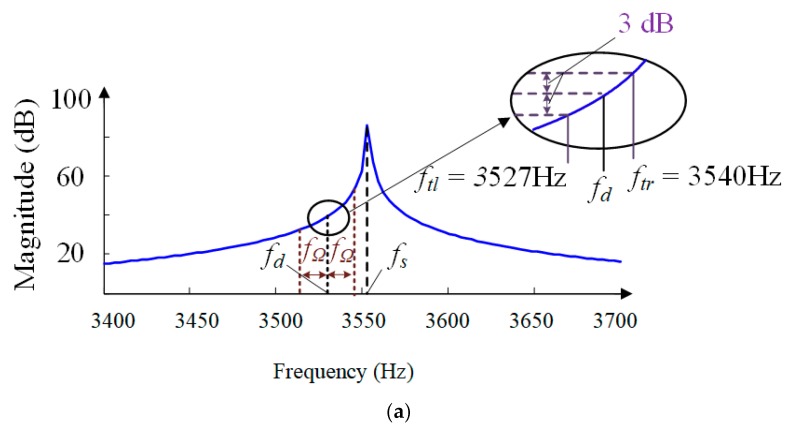

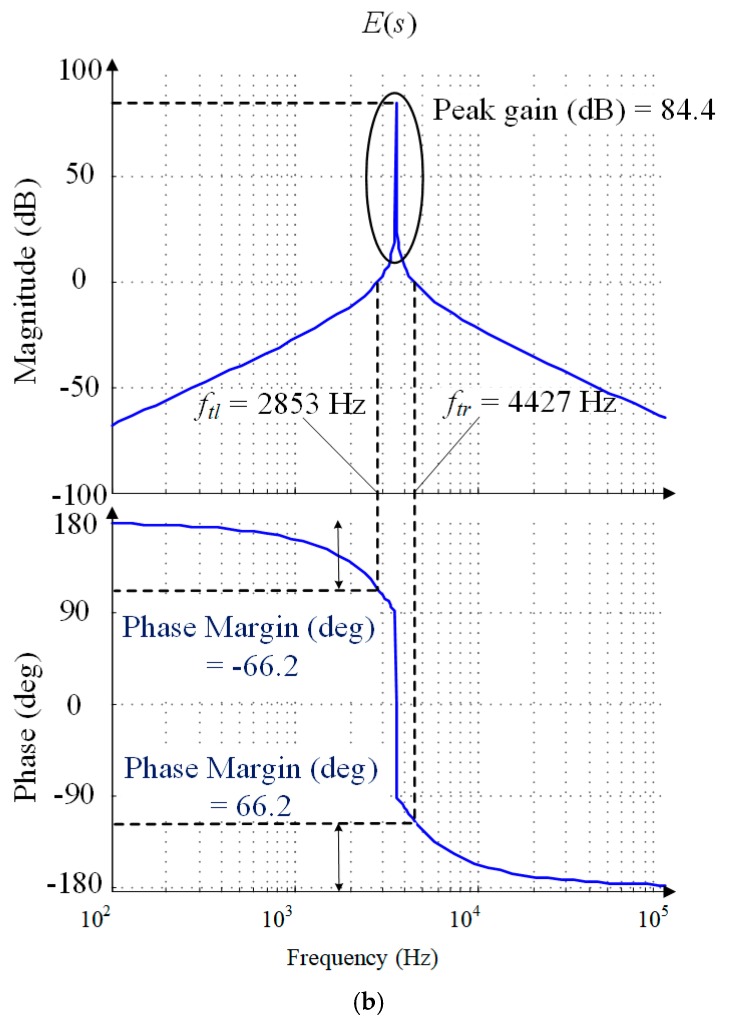
Frequency-domain responses of the open loop system *E*(*s*): (**a**) enlarged view; (**b**) frequency response.

Figure 4 and Figure 5 show the open-loop time-domain and frequency-domain response curves of the sense mode of the MCVG, respectively. When an external disturbance is applied to the sensor, it could be seen in Figure 4a that the sense-mode ringdown oscillation representing the output of the MCVG lasts several seconds, which means that the bias seriously suffers from the external disturbance. Further, the close-up view of the curve is shown in Figure 4b. It can be seen that the setting time is about 9.2 s.

The amplitude-frequency and phase-frequency responses of the sense-mode open-loop function *E*(*s*) are shown in Figure 5. The peak gain of the amplitude-frequency response at the sense-mode resonant frequency reaches 84.4 dB because of the high *Q* of the sense mode. Nevertheless, when the external angular rate with frequency *f*_Ω_ applies to the MCVG, *f*_Ω_ is modulated to a two-side band *f_d_* − *f*_Ω_ and *f_d_* + *f*_Ω_ with the drive-mode resonant frequency *f_d_* of 3535 Hz as the center point, as shown in the detail view; −3 dB bandwidth of the angular rate response is only 13 Hz because of the high *Q*.

Further, the high *Q* will also result in the amplitude response of the angular rate seriously suffering from the fluctuation when the external angular rate varies dynamically with the bandwidth range of 2*f*_Ω_. The corresponding inband spectrum ripple deteriorates to 51.4 dB if the angular frequency *f*_Ω_ varies with the maximum value of 30 Hz. This means that the sense mode itself will inevitably give rise to oscillation and severely deteriorate the performance of the MCVG.

**Figure 6:**
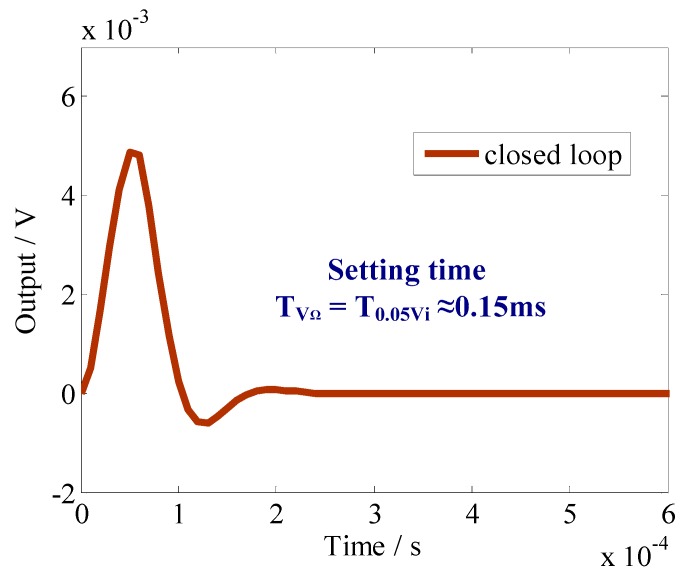
Time-domain response of the sense-mode closed-loop output.

**Figure 7:**
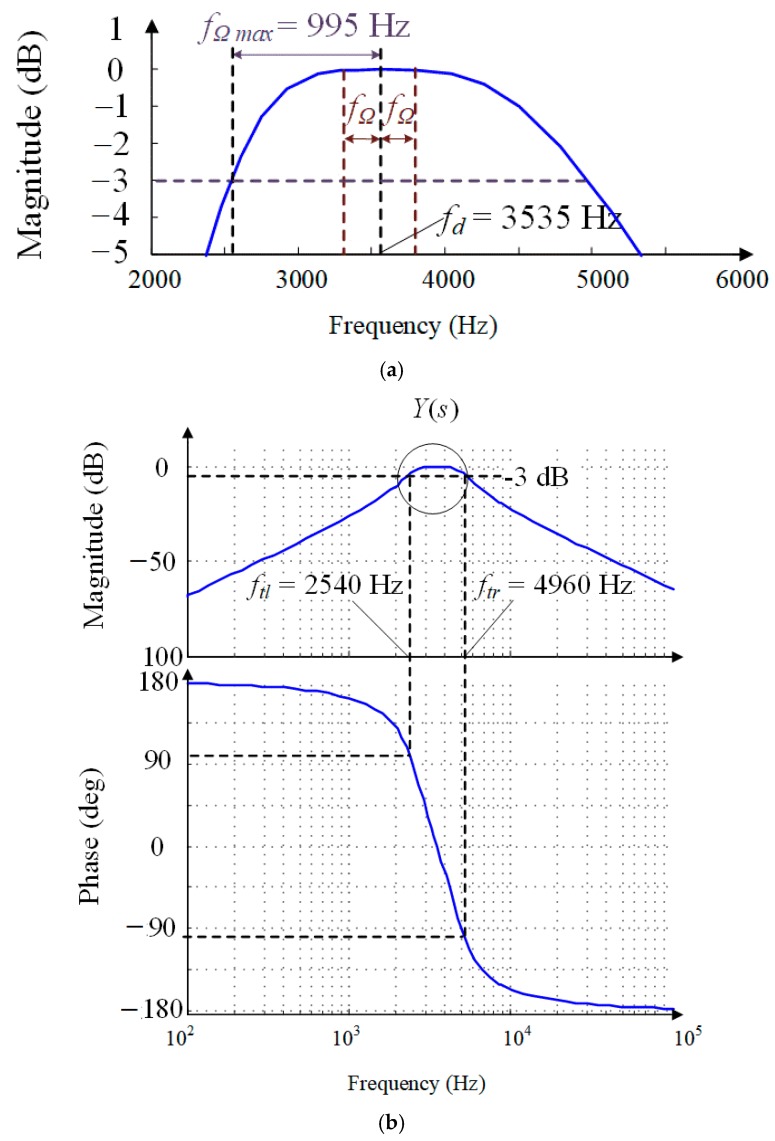
Frequency responses of the closed loop system *Y*(*s*): (**a**) enlarged view; (**b**) frequency response.

In order to improve the response characteristics of the sense mode, the closed-loop oscillation suppression scheme, as described in Figure 2, was simulated as shown in Figure 6 and Figure 7. Figure 6 shows the time-domain response curve of the proposed closed-loop function *Y*(*s*). The setting time is greatly reduced to 0.15 ms.

Further, frequency-domain response curves are shown in Figure 7. It can be seen that −3 dB bandwidth of the angular rate response is increased to 2420 Hz before the demodulation by the proposed control scheme. This bandwidth is sufficiently large so the sense-mode oscillation hardly occurs in the application. Further, the angular-rate amplitude response has the minimum inband spectrum ripple with only 4.23 × 10^−4^ dB. Comparing with the open-loop response, the inband spectrum ripple of the closed-loop control scheme with oscillation suppression is reduced to 2.7‰, which demonstrates that the bias performance of the MCVG can be obviously improved. The comparison results without and with oscillation suppression are listed in Table 3.

It can be seen from Table 3 that the simulated bandwidth of the loop transfer function *Y*(*s*) is 2420 Hz. Nevertheless, this bandwidth is not the actual bandwidth of the whole sense-mode circuit. The whole sense-mode bandwidth is finally determined by the last LPF outside the loop. In detail, the accurate bandwidth of the equivalent band pass filter (BPF) as listed in the above table can be obtained by accurately setting the simulation values of resistor/capacitor (RC) components. Inside the loop, a bandwidth in the range of [*f_d_ − f*_Ω_, *f_d_ + f*_Ω_] before the demodulation signal *V_de_(t)* can be achieved because of the bandwidth of the equivalent BPF. However, after demodulating by reference signal cos(2π*f_d_*) coming from the drive mode, two types of signals with frequencies of *2f_d_* and *f*_Ω_ are generated. By the last LPF outside the loop with a bandwidth of 35 Hz set in this work, a signal with a frequency of *2f_d_* is filtered, but the angular rate signal with a frequency *f*_Ω_ < 35 Hz is retained. Therefore, the whole sense-mode bandwidth is limited to 35 Hz.

## 4. Test Results

**Figure 8:**
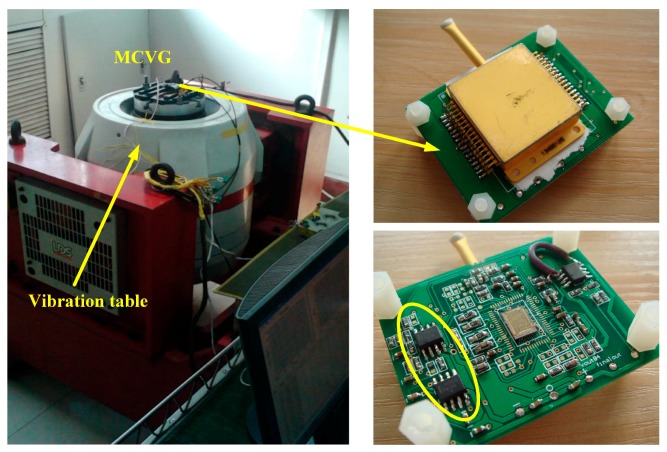
Vibration test system including vibration table and MCVG in vacuum; the signal processing element with oscillation suppression is marked in the yellow circle.

According to the simulations in Section 3, it is demonstrated that the force feedback loop is effective in suppressing the oscillation. A closed-loop circuit was implemented based on the above simulation set, as listed in Table 1 and Table 2. Figure 8 shows the vibration test system consisting of the vibration table, high-Q sensing element, signal processing element application specific integrated circuit (ASIC), and a few discrete RC components, which adjust the key parameters of control electronic elements.

**Figure 9:**
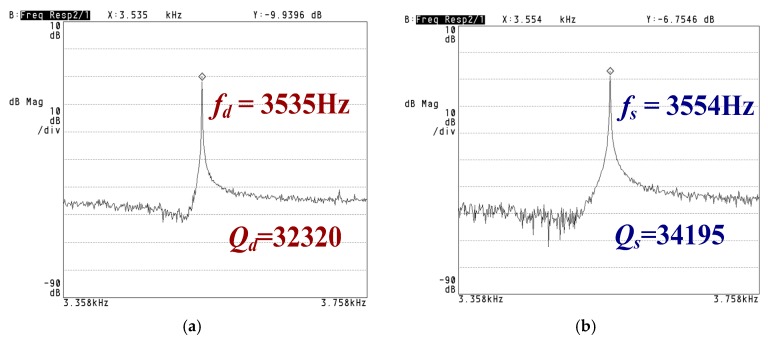
Resonance frequency of the gyroscope: (**a**) drive mode; (**b**) sense mode.

Figure 9 shows that the resonance frequencies of the drive mode and sense mode are measured to be 3535 Hz and 3554 Hz, respectively. The measured results indicate a frequency difference of 19 Hz. The corresponding measured quality factors of the two modes are 32,320 and 34,195, respectively. The corresponding pressure is about 10 Pa.

**Figure 10:**
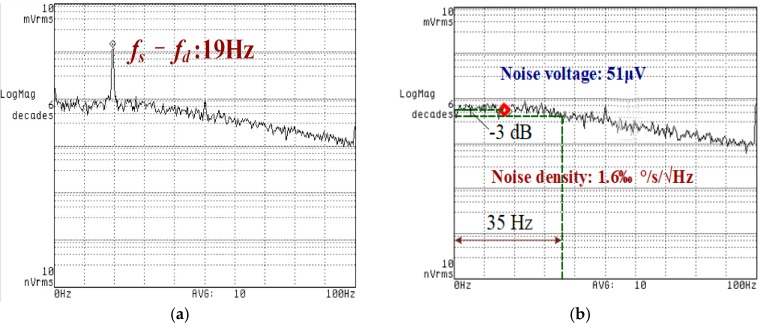
(**a**) Low frequency information generated by external disturbance; (**b**) zero-rate output after oscillation suppression.

The comparison of the frequency-domain spectrum of the zero-rate output without and with oscillation suppression is shown in Figure 10. Within the system bandwidth, it is obvious in Figure 10a that the disturbance signal with a frequency of 19 Hz is about −54 dBV, which implies that the bias stability inevitably suffers from it. Figure 10b shows the noise floor of the gyroscope after oscillation suppression. A disturbance signal of 19 Hz is suppressed. The noise spectrum density of the gyroscope is about 51 µV/√Hz at the setting point of 19 Hz. The equivalent angular rate of the noise power spectral density is 1.6‰ deg/s/√Hz and the bandwidth of the input angular rate signal is 35 Hz, which is mainly determined by the cut-off frequency of the final-stage LPF. The effect of the power frequency of 50 Hz is degraded by properly shielding.

**Figure 11:**
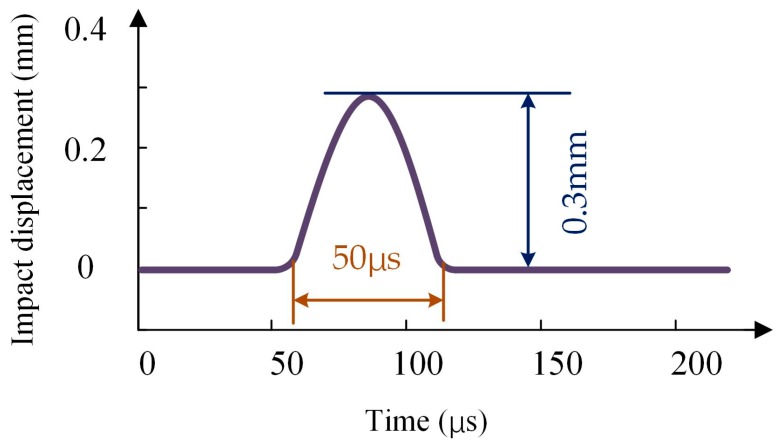
Time-domain impact profile.

The comparison measurement of the oscillation suppression effect in the time domain was performed using the vibration test table shown in Figure 8. The specification of the external disturbance on the measurement is listed in Table 4. The time-domain shock profile is shown in Figure 11.

**Figure 12:**
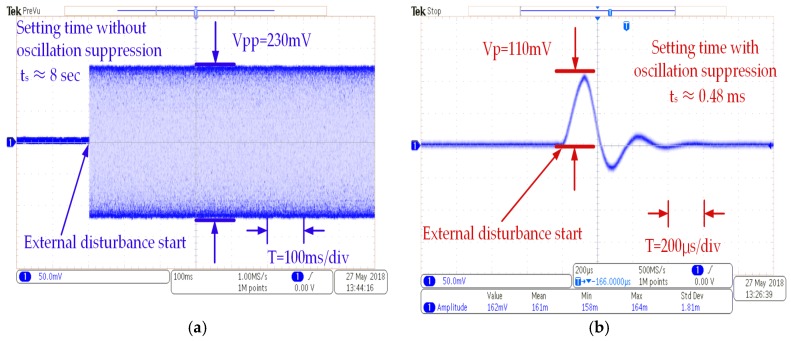
Transient response of the zero angular rate output: (**a**) without and (**b**) with oscillation suppression electronics.

Figure 12a shows that the amplitude of the zero-rate output has a peak-to-peak value of about 230 mV when the external disturbance is applied by the standard vibration table. Assuming a setting time based on the oscillation attenuation curve with an error of 5% of the steady state, the setting time was calculated to be about 8 s. This phenomenon means that the oscillation of the sense mode is nearly in a state of non-damping oscillation with the high *Q*. However, the setting time is greatly shortened to just 0.48 milliseconds with the oscillation suppression circuits as shown in Figure 12b. Compared with the corresponding simulation in Figure 6, the test error of the settling time can contribute to unexpected additional fluctuation of bias output of the MCVG, beyond the fluctuation of the output induced by the external disturbance itself and some potential measurement errors, such as circuit components error and impulse load error caused by assembly.

**Figure 13:**
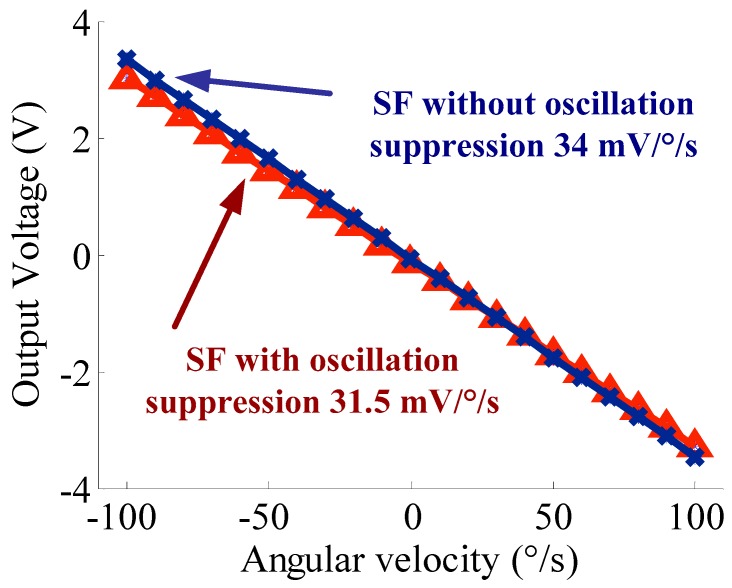
Scale factor comparison of oscillation suppression.

The oscillation effect can also be shown through the scale factor, as shown in Figure 13. The scale factors are 31.5 mV/deg/s and 34 mV/deg/s, respectively. The system sensitivity becomes a slightly lower after the oscillation suppression. The main reason is that the inaccurate RC components result in the fact that the amplitude–frequency response attenuation exists within the frequency domain (*ω_d_* − *ω*_0_, *ω_d_* + *ω*_0_), compared to that of the open-loop detection circuit. Nevertheless, the nonlinearity of the scale factor is reduced to 1.1‰ from 4.5‰.

**Figure 14:**
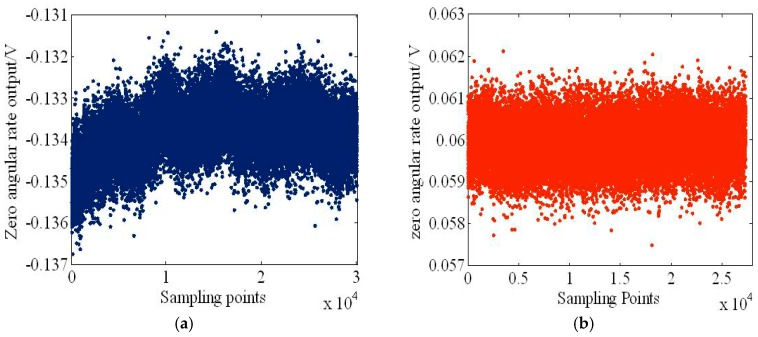
Time slice of the recorded zero angular rate output (**a**) without and (**b**) with oscillation suppression electronics.

Figure 14 shows a slice of the recorded zero angular rate output with and without the oscillation suppression. The sampling period is 100 ms using an Agilent 34401A multi-meter. Figure 14a shows the bias drift curve for 1 hour without the oscillation suppression, which has a bias voltage and bias drift of about 134 mV and 5 mV, respectively. After the oscillation suppression, the bias voltage and bias drift were reduced to 60 mV and 3 mV, respectively, as shown in Figure 14b.

**Figure 15:**
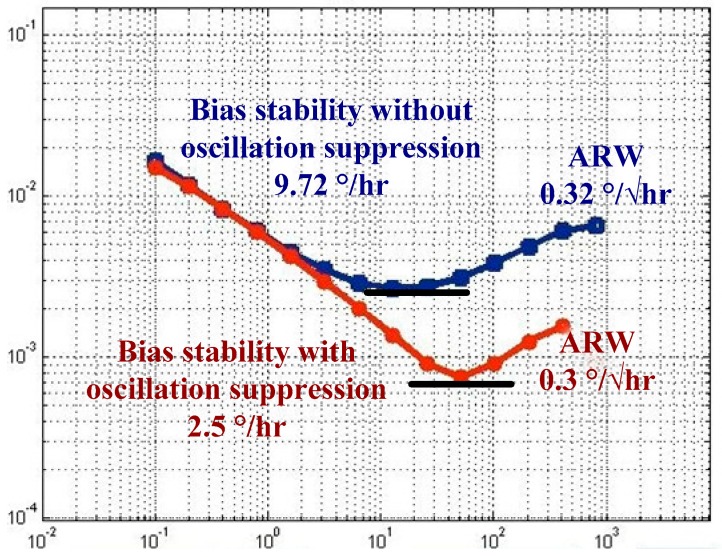
Allan variance curve with and without oscillation suppression.

In Figure 15, the bias stability and angular random walk (ARW) of the gyroscope without the oscillation suppression are 9.72 deg/h and 0.32 deg/√h, respectively. The bias stability and ARW with the oscillation suppression are 2.5 deg/h and 0.3 deg/√h, respectively. This is a significant improvement, and the proposed scheme is thus effective in suppressing the oscillation of the sense mode.

## 5. Conclusions

In this paper, we describe the oscillation of the sense mode for a MCVG with high *Q*. The closed-loop control scheme based on the integrator and HPF is found to greatly suppress this oscillation. Due to no demodulation or modulation modules, the force feedback control circuit is greatly simplified. The experimental results demonstrate that the oscillation suppression scheme is very effective in improving the performance of the MCVG.

## Figures and Tables

**Table 1:** Parameters of the gyroscope.

Gyroscope Parameters	Value
Resonant frequency of the drive mode (*f_d_*)	3535 Hz
Resonant frequency of the sense mode (*f_s_*)	3554 Hz
Mechanical bandwidth	19 Hz
Sense mode mass (*m_s_*)	0.89 mg
Displacement amp. of the drive mode (*X*)	5 μm
Drive-mode quality factor (*Q_d_*)	32,320
Sense-mode quality factor (*Q_s_*)	34,195

**Table 2:** Parameters of the closed-loop system.

Parameters	Value
Gain of displacement-to-voltage interface circuit module (*G_C/V_*)	4 × 10^8^ V/m
Gain of voltage-to-force interface circuit module (*G_V/F_*)	3500 N/V
Gain of integrator (*K_I_*)	100
Cut-off frequency of high pass filter (*f_c_*)	3500 Hz

**Table 3:** Comparison results with and without oscillation suppression.

Gyroscope Parameters	Without Suppression	With Suppression
Setting time of transient response	9.2 s	0.15 ms
Phase margin	66.2 deg	N/A
Cut-off frequency (*f_tl_ f_tr_*)	*f_tl_* = 3527 Hz *f_tr_* = 3540 Hz	*f_tl_* = 2540 Hz *f_tr_* = 4960 Hz
Bandwidth	13 Hz	2420 Hz
Inband spectrum ripple	51.4 dB	4.23 × 10^−4^ dB

**Table 4:** Specification of external disturbance.

Parameters of Impulse Test	Value
Equivalent weight of movable platform	2000 g
Shock displacement	0.3 mm
Impact acceleration	490 m/s^2^
pulse duration	50 μs
